# The Effects of Bariatric Surgery Weight Loss on Knee Pain in Patients with Osteoarthritis of the Knee

**DOI:** 10.1155/2012/504189

**Published:** 2012-12-03

**Authors:** Christopher Edwards, Ann Rogers, Scott Lynch, Tamara Pylawka, Matthew Silvis, Vernon Chinchilli, Timothy Mosher, Kevin Black

**Affiliations:** ^1^Penn State College of Medicine, Penn State Hershey Medical Center, Penn State University, 500 University Drive, Hershey, PA 17033, USA; ^2^Department of Surgery, Penn State Hershey Medical Center, 500 University Drive, MC H149, Hershey, PA 17033, USA; ^3^Department of Orthopaedics and Rehabilitation, Penn State Hershey Medical Center, 30 Hope Drive, P.O. Box 859, Hershey, PA 17033, USA; ^4^Department of Family and Community Medicine, Penn State Hershey Medical Center, 500 University Drive, H154, Hershey, PA 17033, USA; ^5^Department of Health Evaluation Sciences, Penn State College of Medicine, Penn State Hershey Medical Center, 600 Centerview Drive, Suite 2200, P.O. Box 855, Hershey, PA 17033, USA; ^6^Department of Radiology, Penn State Hershey Medical Center, 500 University Drive, H066, Hershey, PA 17033, USA

## Abstract

Studies have shown that osteoarthritis (OA) is highly associated with obesity, and individuals clinically defined as obese (BMI > 30.0 kg/m^2^) are four times more likely to have knee OA over the general population. The purpose of this research was to examine if isolated weight loss improved knee symptoms in patients with osteoarthritis. Adult patients (*n* = 24; age 18–70; BMI > 35 kg/m^2^) with clinical and radiographic evidence of knee OA participated in a one-year trial in which WOMAC and KOOS surveys were administered at a presurgery baseline and six and twelve months postsurgery. Statistical analysis was performed using Student's *t* and Wilcoxon Signed Rank tests. Weight loss six and twelve months following bariatric surgery was statistically significant (*P* < 0.05) compared to presurgery measurements. All variables from both KOOS and WOMAC assessments were significantly improved (*P* < 0.05) when compared to baseline. Isolated weight loss occurring via bariatric surgery resulted in statistically significant improvement in patient's knee arthritis symptoms at both six and twelve months. Further research will need to be done to determine if symptom relief continues over time, and if the benefits are also applicable to individuals with symptomatic knee arthritis that are overweight but not obese.

## 1. Introduction

Osteoarthritis (OA) of the knee is one of the five leading causes of disability among elderly men and women in the United States [1]. In 2008, an estimated 36 million ambulatory care visits were reported due to knee-related complaints [2], while from 1995–2005 the number of individuals with OA increased by approximately six million. Kotlarz et al. estimated the combined insurer and out-of-pocket healthcare expenditures of OA to be $185 billion [3].

Obesity is one of the leading risk factors for the development of knee OA, and population studies show that the increased incidence of obesity in the USA has correlated with a similar rise in knee osteoarthritis. In 2005-2006 more than 33% of the USA population was obese [4, 5], a dramatic increase from 1980 when estimates were near 15%. Individuals clinically defined as obese (BMI > 30.0 kg/m^2^) are four times more likely to have knee OA than those with a BMI < 25.0 kg/m^2^ [6]. Even at the upper level of a normal BMI, (22–25 kg/m^2^), there has been shown to be an increased risk in developing medial compartment and patellofemoral knee OA [7].

Although it has been well established that obesity and being overweight are risk factors for the development of osteoarthritis, the literature is sparse when investigating the impact of weight loss on arthritis symptoms. Messier et al. used various combinations of diet and exercise as weight loss approaches to investigate the impact of weight reduction on knee pain and found that although neither diet nor exercise alone significantly improved knee pain, the two together did produce significant improvements [8]. Christensen et al. reported improved knee pain and function in patients who lost 10% of their body weight [9]. Based on published material, weight loss via diet and exercise produces significant improvements in knee pain and physical function, but the question of whether the hypocaloric diet or the exercise regimen is the most influential in producing the improvements on knee pain, to the best of our knowledge, remains unanswered. 

We hypothesized that weight reduction, via bariatric surgery and in the absence of any other arthritis treatment, would result in significant improvements in the symptoms associated with knee arthritis. Bariatric surgery allows weight loss to occur independently from potentially confounding variables involved in diet and exercise and we believed would give us a more accurate depiction of the effects of weight loss on knee pain and related functions.

## 2. Methods

### 2.1. Study Participants

After receiving institutional IRB approval, potential study candidates were identified by one of the investigators (AMR) who would be performing the bariatric surgery. Inclusion criteria for bariatric surgery included being between the age of 18 and 70, having a body mass index (BMI) of at least 35 kg/m^2^, and passing a psychological evaluation. Potential participants were then screened for symptoms of knee arthritis of greater than one-year duration utilizing the Western Ontario and McMaster Universities (WOMAC) Index of Osteoarthritis. Those that reported at least intermittent knee pain for the prior 12-month period and had a WOMAC pain score between 3 and 7 were then asked to be evaluated by an orthopaedic physician and have X-rays taken of their knees. Standing AP, LAT, and Merchant radiographs were used to evaluate the knee for the presence of osteoarthritis. A board certified radiologist scored the degree of arthritis present on X-ray using the Kellgren and Lawrence scoring system [10]. In this radiographic classification system, a score of 0 indicates normal X-rays, 1 signifies minute osteophytes of doubtful clinical significance, 2 indicates definite osteophytes with unimpaired joint space, while 3 and 4 also demonstrate moderate joint space narrowing (3) and severe joint space narrowing and subchondral sclerosis (4). A physical exam was conducted by an orthopaedic surgeon. Only individuals with both clinical and radiographic evidence of knee osteoarthritis were allowed to participate in the study (*n* = 24). 

Individuals were excluded from the study if they were unwilling to have X-rays taken of their knees or be examined by a physician. Finally, the subset of patients who have had previous total knee arthroplasty who were currently taking prescription nonsteroidal anti-inflammatory medications for pain or receiving viscosupplementation or corticosteroid injections were excluded from the study. Patients using other oral pain relieving medications such as acetaminophen were not excluded from the study as they have not been shown to have a direct physiologic effect on knee arthritis.

### 2.2. Study Design

The two health status measurement questionnaires used in this study were the Western Ontario and McMaster Universities (WOMAC) Index of Osteoarthritis and the Knee Osteoarthritis Outcome Score (KOOS). Both methods have been previously validated in the literature [11–13]. The WOMAC is a self-administered survey which specifically targets symptoms of pain, stiffness, and physical function. It consists of 24 questions (5 pain, 2 stiffness, and 17 physical function). The KOOS is a similar instrument which was intended to be used to assess the development or progression of knee osteoarthritis. It consists of five subscales including pain and other symptoms associated with arthritis, function in daily living and recreation, and quality of life. Each study participant completed WOMAC and KOOS surveys prior to their bariatric surgery as a baseline measurement of knee pain and disability. Repeat questionnaires were mailed to study participants six and twelve months after surgery which were completed and returned in a prepaid envelope. Format used for scoring in both KOOS and WOMAC was 100 mm visual analogue.

Three bariatric surgery techniques were used on study participants. Eighteen patients had roux-en-Y gastric bypass surgery performed, four patients underwent Realize Band placement, and one had a sleeve gastrectomy. Patient body weight was recorded prior to surgery and at regular intervals after. Patient weights at six and twelve months postsurgery were used in this study. The same weight measurement scale was used for all patient visits, and the device was calibrated on regular intervals. At each interval, BMI was calculated based on patient height and weight.

### 2.3. Statistical Analysis

Paired statistical tests were applied on the data collected in order to detect a change in the WOMAC and KOOS scores from baseline to six and twelve months. Statistical analysis was performed using data from all subjects. Student's *t* test and Wilcoxon Signed Rank tests were used to determine the *P* values. Correlation analysis using the CORR procedure was done to compare changes in weight and Body Mass Index to changes in KOOS and WOMAC scores. The analyses were completed using the SAS System (“Local”, XP_PRO).

## 3. Results

Between September 2008 and May 2009, 54 patients were scheduled for bariatric surgery by one of the coinvestigators. Thirty patients were excluded as they did not meet inclusion criteria, while 24 met criteria and agreed to participate. 

The degree of osteoarthritis present in the knee for all patients was measured radiographically using the Kellgren and Lawrence scoring system [10]. The average score for the right knee was 1.89, and the average score for the left was 1.85. 

**Figure 1:**
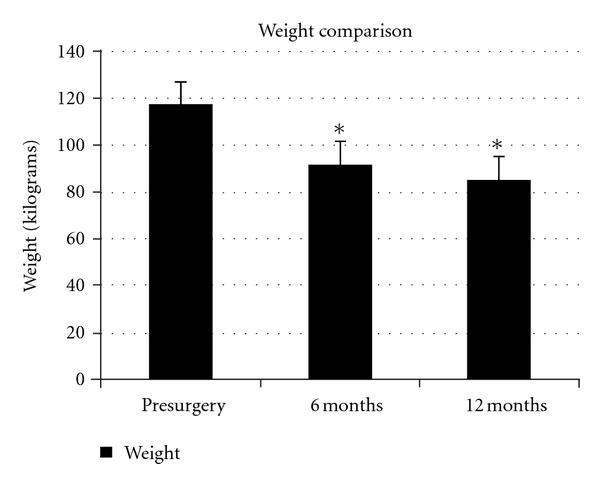
Comparison of body weight from baseline (presurgery) to 12 month postsurgery follow-up appointment. Standard error bars are present. The (∗) indicates significant weight loss at 6 months and 12 months when compared to baseline. *n* = 24.

**Figure 2:**
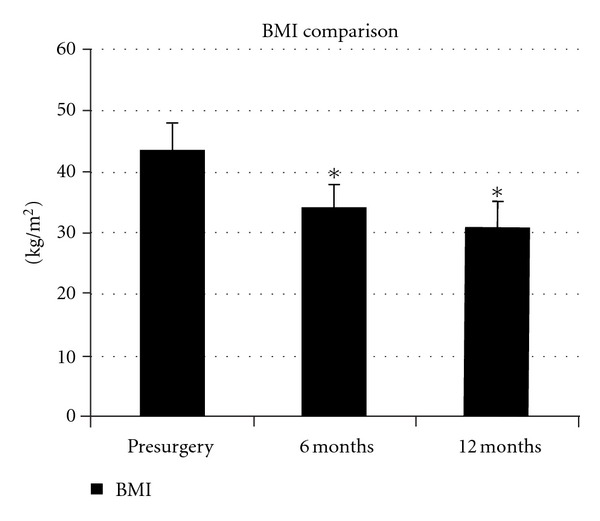
Comparison of Body Mass Index (BMI) from baseline (pre-surgery) to 12 month postsurgery follow-up appointment. Standard error bars are present. The (∗) indicates significant weight loss at 6 months and 12 months when compared to baseline. *n* = 24.

The mean preoperative weight of the patients was 117.7 kg, and the average weight loss per patient was 25.9 kg (*P* < 0.0001) at 6 months postsurgery and 32.4 kg (*P* < 0.0001) at 12 months (Figure 1). The average change in BMI was −9.60 (*P* < 0.0001) at 6 months and −12.6 (*P* < 0.0001) at 12 months (Figure 2). There was one study participant who gained weight following the procedure (112.7 to 115 kg). There were three other study participants who lost minimal weight. One patient lost 3.2 kg from 87.7 to 84.5 kg while another lost 3.6 kg from 115 to 111.4 kg. The third individual decreased from 143.6 to 137.3 kg, a loss of 6.4 kg. All 4 of the individuals not losing a significant amount of weight had undergone Realize Band placement and were felt by us to have an unsuccessful surgical outcome.

**Figure 3:**
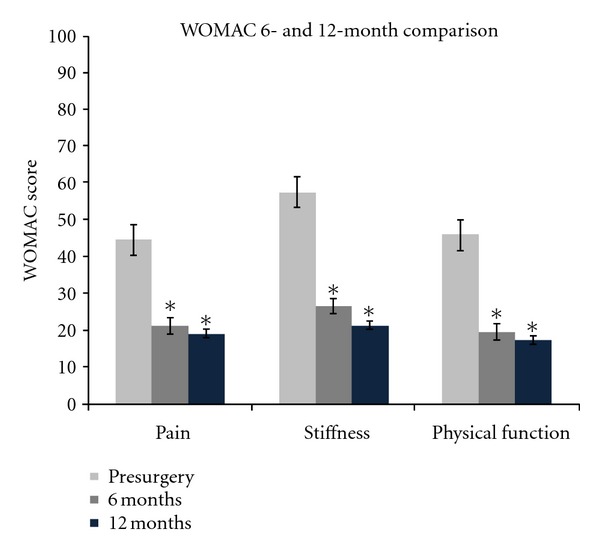
WOMAC pain, stiffness, and physical function comparisons from baseline (pre-surgery) to 12 month follow-up. The bars are standard error. The (∗) indicates significant differences (*P* < 0.05) when compared to baseline. *n* = 24.

WOMAC scores for all participants were compared at baseline and at 6 and 12 months postsurgery. The change from baseline showed significant improvement (*P* < 0.05) in all variables measured including pain, stiffness, and physical function.  Figure 3 shows the change in WOMAC variables from baseline to 12 months postsurgery.

**Figure 4:**
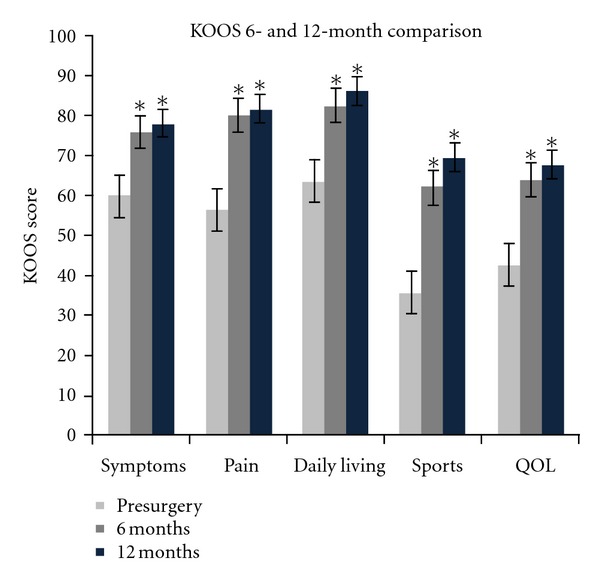
Comparison between KOOS scores reported at baseline and then 6- and 12-month postsurgery. Bars are standard error. The (∗) indicates significance (*P* < 0.05). *n* = 24. QOL = quality of life.

Findings with the KOOS scores were similar. There was a significant improvement (*P* < 0.05) in all categories measured including pain, stiffness, other symptoms, activities of daily living, sports, and quality of life.  Figure 4 shows the change from baseline across all variables. Refer to Tables 1 and 2 for statistical analysis including mean, median, and first/third quartiles for WOMAC and KOOS scores comparing baseline to 6 and 12 months postsurgery.

In order to investigate the relationship between changes in weight and BMI to changes in KOOS and WOMAC scores, a Pearson Correlation analysis was performed which showed a positive correlation between all variables (see Table 3).

## 4. Discussion

We have demonstrated that isolated weight loss occurring via bariatric surgery has resulted in statistically significant improvement in patient's knee arthritis symptoms at both six and twelve months following bariatric surgery. Pain, stiffness, and physical function were knee specific parameters and showed marked improvement. Other factors such as quality of life, activities of daily living, and sports activities also showed highly significant improvements.

Pearson correlation analysis shows positive correlation between changes in KOOS/WOMAC scores and change in weight/BMI. This demonstrates that there is a trend towards weight loss being directly linked with improvement in knee OA symptoms in the absence of other variables. Small sample size and one-year followup make achieving statistical significance difficult to achieve with a correlation analysis. 

The current treatment recommendations for knee arthritis according to the OA Research Society International (OARSI) include more than 50 different treatment modalities, most of which are conservative in nature. Only after the failure of conservative management should surgical intervention be pursued. Zhang et al. reviewed various nonsurgical treatment modalities including NSAIDs, physical therapy, bracing, steroid injections, viscosupplementation, and weight reduction. They showed that weight reduction can significantly improve symptoms related to knee arthritis. Most of the studies included in this paper looked at weight loss in combination with other confounding variables such as NSAIDs and steroid injections [14].

While other research has been done to determine if weight loss in combination with other treatments can improve knee pain, we believe that the current study is one of the few to demonstrate that weight loss, by itself, results in significant improvement in knee pain and stiffness in patients with radiographic evidence of arthritis. 

Messier et al. evaluated 252 obese and overweight patients over the age of 60 who had knee pain and radiographic evidence of knee OA over a period of 18 months [8]. Patients were randomized into either diet alone, exercise alone, diet and exercise, or healthy lifestyle groups (control). They found that diet and exercise together resulted in significant overall improvements in knee pain according to the WOMAC, whereas diet alone and exercise alone did not produce significant improvements in knee pain [8].

Christensen et al. studied the effects of isolated weight loss on knee pain using a low-energy diet, 3.4 MJ/day, as their only form of caloric intake [9]. Using the WOMAC, Christensen found that the total WOMAC score, pain score, and physical function subscale were significantly improved. The study concluded that in patients with knee OA, a weight reduction of 10% improved physical function by 28% [9]. 

Miller et al. evaluated 87 obese patients over the age of 60 with knee pain and difficulty with daily activities. They were treated with a 1000 kcal/day diet and an intense physical exercise program over a period of six months. They reported that all subscales from the WOMAC (pain, stiffness, and physical function) had significant improvement from baseline (*P* < 0.05) [4]. However, physical exam diagnosis and X-ray confirmation of knee OA were not utilized in their investigation, making it difficult to determine if the improvements they were finding were actually in patients with knee OA. 

Hooper et al. evaluated 48 obese individuals greater than 35 years of age who had a history of knee pain and physical findings suggestive of OA [7]. The combination of bariatric surgery weight loss and 30 minutes of physical activity three days a week were utilized to investigate changes in knee pain. Their results showed significant improvement in all categories of WOMAC and Short Form 36 (*P* < 0.05) [7]. OA was not radiographically confirmed, and weight loss was not an independent factor as both bariatric surgery and exercise were used in the protocol. 

Peltonen et al. compared 1135 random samples from the general population to 6325 obese individuals from the Swedish obese subjects (SOSs) study with regard to musculoskeletal pain including knee pain [15]. Patients were included if they had pain that periodically restricted their working capacity during the past 12 months. Obese individuals treated with gastric banding, vertical banded gastroplasty, or gastric bypass were found to have greater knee pain reduction compared to individuals treated with conservative weight loss and to weight neutral controls. Drawbacks to this study included the lack of clinical and radiographic evidence of knee osteoarthritis and lack of standardized pain questionnaires (i.e., WOMAC, KOOS, and Short Form) used to quantify the knee pain. 

We believe that exercise is an important part of health and can contribute to weight loss efforts, but our results suggest that weight loss, independent of other factors such as diet and exercise, is most likely the underlying factor responsible for the statistically significant improvement in knee pain and related symptoms.

The current first-line treatment approach for patients with knee OA includes modification of activity, over the counter analgesics, prescription anti-inflammatory medications (NSAIDs), cortisone injections and viscosupplementation, arthroscopy, and knee arthroplasty. While NSAIDs have been shown to temporarily relieve the pain and swelling associated with OA, there are several negative side-effects related to NSAID use. The most common side-effect of NSAID use has been gastrointestinal upset with daily symptoms occurring in 16% of individuals according to Laine [16]. Studies have also found that long-term NSAID use has been associated with emergency department visits secondary to upper gastrointestinal problems [17]. Hospital records confirm nearly 65,000 admissions each year in the United Kingdom for upper GI-related symptoms and attribute 12,000 of those admissions to NSAID use (including 2,230 deaths) [17–19]. In hope of reducing gastrointestinal side-effects, the COX-2 inhibitor class of NSAIDs has been used extensively for the treatment of OA since their introduction to the USA in 1997. This is evidenced by the rise in total spending on COX-2 inhibitors from zero in 1997 to $5.5 billion in 2003 [20]. However, increased risk of myocardial infarction was linked to specific COX-2 inhibitors [21], while other NSAIDs have been shown to cause blood pressure elevation compared with placebo [22].

Intraarticular hyaluronate (IAH) or viscosupplementation has been shown to benefit many patients with symptomatic knee OA and has become an increasingly popular treatment for those with knee OA [23, 24]. The American College of Rheumatology recommends using viscosupplementation when other nonsurgical therapies such as physical therapy and analgesics have failed or when NSAIDs are contraindicated. Watterson estimates that a series of injections can cost $500 per knee [24]. 

 Although the role of arthroscopy in the treatment of patients with knee arthritis is controversial, it has been reported to be the most common surgery performed for knee OA. Moseley stated that over 650,000 arthroscopic lavage or debridement procedures occur each year in the USA for patients when medical therapy fails to provide adequate pain relief for knee arthritis. The average expense per knee arthroscopy reported was $5,000 [25]. 

The number of knee arthroplasty procedures has been increasing in the United States. Utilizing National Inpatient Sample Data from 1990 to 2003 and United States Census Bureau data, Kurtz et al. estimate that by 2030 there will be an expected 670% increase in the number of TKRs performed [26]. In 2005 there were almost 500,000 TKRs performed in the USA, with an annual expenditure surpassing $11 billion [6]. 

According to the NIH, weight loss surgery has an average per patient cost of $12,000 and $35,000. More work will need to be done in order to determine the cost effectiveness of weight loss surgery over the current treatment modalities for knee arthritis.

## 5. Conclusions

The need for cost containment in the American health care system has been discussed for decades. This study evaluates a specific group of obese individuals that are undergoing bariatric surgery. There are many other patients that are overweight and obese but not candidates for this procedure. The obesity epidemic is certainly multifactorial in etiology. Although commercial payers in central Pennsylvania will provide coverage for medications, injections, arthroscopy, and knee arthroplasty, they do not routinely provide financial coverage for meaningful nutritional counseling. We believe this is a major gap in our treatment of this condition, and we hope to pursue this further in another investigation. We believe comparison can be drawn to our treatment algorithm for patients with osteoporosis and fragility fractures. When a patient is known to have low bone mass, we realize that they are at risk for the development of a fragility fracture, and via medication and exercise treat the osteoporosis. Yet when overweight or obese patients present with symptomatic knee arthritis we typically treat the arthritis but not the underlying disease. We believe that rectifying this approach can play a meaningful role in joint health in the years ahead, while at the same time improving the overall health of the patient and potentially saving health care dollars in the future.

In summary, we have shown that patients with symptomatic knee arthritis who undergo bariatric surgery show improvement in knee pain and other symptoms as reported in KOOS and WOMAC questionnaires. We believe it will be important to follow them on a longer term basis to see if their symptom improvement continues over time. Finally, future research on this topic should focus on the effects of weight loss in not only the obese but overweight individuals (BMI = 25–29.9).

## Figures and Tables

**Table 1:** Statistical analysis of KOOS and WOMAC scores comparing values at baseline and 6 months. Values listed are the change in scores over a 6-month period.

KOOS and WOMAC scores: baseline to 6-month comparison					
	Mean	Q1	Median	Q3	Signed rank *P* value
WOMAC pain	−4.83	−7.29	−5.2	−3.12	<0.0001
WOMAC stiffness	−2.33	−4.17	−3.13	0	0.0001
WOMAC physical function	−17.76	−28.13	−17.71	−8.34	<0.0001
KOOS symptoms	8.64	0	8	12	0.0003
KOOS pain	17.78	4.44	17.78	31.11	<0.0001
KOOS daily living	13.43	5.29	11.76	24.7	<0.0001
KOOS sports	15.76	0	12	32	0.001
KOOS QOL	15.8	5	15	30	<0.0001

Q1: 1st quartile, Q3: 3rd quartile, QOL: quality of life. For all categories *n* = 24. Values listed above are measured as differences in WOMAC/KOOS scores subtracted from baseline.

**Table 2:** Statistical analysis of KOOS and WOMAC scores comparing values at baseline and 12 months. Values listed are the change in scores over a 12-month period

KOOS and WOMAC scores: baseline to 12-month comparison.					
	Mean	Q1	Median	Q3	Signed rank *P* value
WOMAC pain	−5.295	−7.29	−6.25	−4.685	<0.0001
WOMAC stiffness	−2.95	−4.17	−3.645	−2.085	<0.0001
WOMAC physical function	−19.2	−31.775	−20.84	−11.46	<0.0001
KOOS symptoms	11.66	4	12	16	<0.0001
KOOS pain	19.44	7.775	18.89	28.33	<0.0001
KOOS daily living	17.36	7.65	14.12	26.62	<0.0001
KOOS sports	22.43	8	20	32	<0.0001
KOOS quality of life	18.5	10	17.5	27.5	<0.0001

Q1: 1st quartile, Q3: 3rd quartile. For all categories *n* = 24. Values listed above are measured as differences in WOMAC/KOOS scores subtracted from baseline.

**Table 3:** Pearson correlation analysis.

Parameter		Weight change at 6 months	Weight change at 12 months
KOOS symptoms	Correlation coefficient	0.14768	0.07676
	Sig (two tailed)	0.5229	0.7621
	*n*	21	18
KOOS pain	Correlation coefficient	−0.0697	−0.15328
	Sig (two tailed)	0.764	0.5437
	*n*	21	18
KOOS daily living	Correlation coefficient	0.14679	−0.10796
	Sig (two tailed)	0.5255	0.9436
	*n*	21	18
KOOS sports	Correlation coefficient	−0.162554	−0.38754
	Sig (two tailed)	0.4815	0.1121
	*n*	21	18
KOOS quality of life	Correlation coefficient	0.22804	0.19418
	Sig (two tailed)	0.3201	0.4401
	*n*	21	18
WOMAC pain	Correlation coefficient	−0.06586	0.11497
	Sig (two tailed)	0.7767	0.6496
	*n*	21	18
WOMAC stiffness	Correlation coefficient	−0.025	0.1714
	Sig (two tailed)	0.9144	0.4965
	*n*	21	18
WOMAC physical function	Correlation coefficient	−0.12644	−0.12462
	Sig (two tailed)	0.585	0.6222
	*n*	21	18

Row one for each parameter shows the pearson correlation Coefficient. Row two is Rho > *r* under HO: Rho = 0. There was *n* = 21 at 6 months and *n* = 18 at 12 months.

## References

[1] Ong KL, Mowat FS, Chan N, Lau E, Halpern MT, Kurtz SM (2006). Economic burden of revision hip and knee arthroplasty in medicare enrollees. *Clinical Orthopaedics and Related Research*.

[2] Helmick CG, Felson DT, Lawrence RC (2008). Estimates of the prevalence of arthritis and other rheumatic conditions in the United States. Part I. *Arthritis and Rheumatism*.

[3] Kotlarz H, Gunnarsson CL, Fang H, Rizzo JA (2009). Insurer and out-of-pocket costs of osteoarthritis in the US: evidence from national survey data. *Arthritis and Rheumatism*.

[4] Miller GD, Nicklas BJ, Davis C, Loeser RF, Lenchik L, Messier SP (2006). Intensive weight loss program improves physical function in older obese adults with knee osteoarthritis. *Obesity*.

[5] Ogden CL, Carroll MD, McDowell MA, Flegal KM (2007). Obesity among adults in the United States—no change since 2003-2004. *NCHS Data Brief*.

[6] Losina E, Walensky RP, Kessler CL (2009). Cost-effectiveness of total knee arthroplasty in the United States: patient risk and hospital volume. *Archives of Internal Medicine*.

[7] Hooper MM, Stellato TA, Hallowell PT, Seitz BA, Moskowitz RW (2007). Musculoskeletal findings in obese subjects before and after weight loss following bariatric surgery. *International Journal of Obesity*.

[8] Messier SP, Loeser RF, Miller GD (2004). Exercise and dietary weight loss in overweight and obese older adults with knee osteoarthritis: the arthritis, diet, and activity promotion trial. *Arthritis and Rheumatism*.

[9] Christensen R, Astrup A, Bliddal H (2005). Weight loss: the treatment of choice for knee osteoarthritis? A randomized trial. *Osteoarthritis and Cartilage*.

[10] Kijowski R, Blankenbaker D, Stanton P, Fine J, De Smet A (2006). Arthroscopic validation of radiographic grading scales of osteoarthritis of the tibiofemoral joint. *American Journal of Roentgenology*.

[11] Angst F, Aeschlimann A, Steiner W, Stucki G (2001). Responsiveness of the WOMAC osteoarthritis index as compared with the SF-36 in patients with osteoarthritis of the legs undergoing a comprehensive rehabilitation intervention. *Annals of the Rheumatic Diseases*.

[12] Bellamy N, Buchanan WW, Goldsmith CH, Campbell J, Stitt LW (1988). Validation study of WOMAC: a health status instrument for measuring clinically important patient relevant outcomes to antirheumatic drug therapy in patients with osteoarthritis of the hip or knee. *Journal of Rheumatology*.

[13] Roos EM, Toksvig-Larsen S (2003). Knee injury and Osteoarthritis Outcome Score (KOOS)—validation and comparison to the WOMAC in total knee replacement. *Health and Quality of Life Outcomes*.

[14] Zhang W, Nuki G, Moskowitz RW (2010). OARSI recommendations for the management of hip and knee osteoarthritis. Part III: Changes in evidence following systematic cumulative update of research published through January 2009. *Osteoarthritis and Cartilage*.

[15] Peltonen M, Lindroos AK, Torgerson JS (2003). Musculoskeletal pain in the obese: a comparison with a general population and long-term changes after conventional and surgical obesity treatment. *Pain*.

[16] Laine L (2006). GI risk and risk factors of NSAIDs. *Journal of Cardiovascular Pharmacology*.

[17] Blower AL, Brooks A, Fenn GC (1997). Emergency admissions for upper gastrointestinal disease and their relation to NSAID use. *Alimentary Pharmacology and Therapeutics*.

[18] Moore RA, Phillips CJ (1999). Cost of NSAID adverse effects to the UK national health service. *Journal of Medical Economics*.

[19] Tarone RE, Blot WJ, McLaughlin JK (2004). Nonselective nonaspirin nonsteroidal anti-inflammatory drugs and gastrointestinal bleeding: relative and absolute risk estimates from recent epidemiologic studies. *American Journal of Therapeutics*.

[20] Banthin JS, Zodet M (2006). Trends in the use and expenditures for COX-2 inhibitors and traditional nonsteroidal anti-inflammatory drugs, 1997–2003. *Statistical Brief*.

[21] Graham GG, Graham RI, Day RO (2002). Comparative analgesia, cardiovascular and renal effects of celecoxib, rofecoxib and acetaminophen (paracetamol). *Current Pharmaceutical Design*.

[22] Aw TJ, Haas SJ, Liew D, Krum H (2005). Meta-analysis of cyclooxygenase-2 inhibitors and their effects on blood pressure. *Archives of Internal Medicine*.

[23] Bellamy N, Campbell J, Robinson V, Gee T, Bourne R, Wells G (2006). Viscosupplementation for the treatment of osteoarthritis of the knee. *Cochrane Database of Systematic Reviews*.

[24] Watterson JR, Esdaile JM (2000). Viscosupplementation: therapeutic mechanisms and clinical potential in osteoarthritis of the knee. *The Journal of the American Academy of Orthopaedic Surgeons*.

[25] Bruce Moseley J, O’Malley K, Petersen NJ (2002). A controlled trial of arthroscopic surgery for osteoarthritis of the knee. *The New England Journal of Medicine*.

[26] Kurtz S, Ong K, Lau E, Mowat F, Halpern M (2007). Projections of primary and revision hip and knee arthroplasty in the United States from 2005 to 2030. *Journal of Bone and Joint Surgery A*.

